# Emergence of a Latent Indian Cassava Mosaic Virus from Cassava Which Recovered from Infection by a Non-Persistent Sri Lankan Cassava Mosaic Virus

**DOI:** 10.3390/v8100264

**Published:** 2016-09-28

**Authors:** Chockalingam Karthikeyan, Basavaprabhu L. Patil, Basanta K. Borah, Thulasi R. Resmi, Silvia Turco, Mikhail M. Pooggin, Thomas Hohn, Karuppannan Veluthambi

**Affiliations:** 1 Department of Plant Biotechnology, School of Biotechnology, Madurai Kamaraj University, Madurai-625021, Tamil Nadu, India; karthikbiomdu@gmail.com (C.K.); trresmi@gmail.com (T.R.R.); kveluthambi@rediffmail.com (K.V.); 2 Institute of Botany, University of Basel, Schöenbeinstrasse 6, Basel 4056, Switzerland; basavaprabhu.patil@icar.gov.in (B.L.P.); basantabora@gmail.com (B.K.B.); silvia.turco@unibas.ch (S.T.); Mikhail.Pooggin@unibas.ch (M.M.P.); 3 Present address: ICAR-National Research Centre on Plant Biotechnology, PusaCampus, New Delhi110012, India; 4 Present address: Department of Agricultural Biotechnology, Assam Agricultural University, Jorhat 785013, India

**Keywords:** cassava, geminivirus, persistent and non-persistent SLCMV, ICMV, pseudo-recombination, *trans*-replication

## Abstract

The major threat for cassava cultivation on the Indian subcontinent is cassava mosaic disease (CMD) caused by cassava mosaic geminiviruses which are bipartite begomoviruses with DNA A and DNA B components. Indian cassava mosaic virus (ICMV) and Sri Lankan cassava mosaic virus (SLCMV) cause CMD in India. Two isolates of SLCMV infected the cassava cultivar Sengutchi in the fields near Malappuram and Thiruvananthapuram cities of Kerala State, India. The Malappuram isolate was persistent when maintained in the Madurai Kamaraj University (MKU, Madurai, Tamil Nadu, India) greenhouse, whereas the Thiruvananthapuram isolate did not persist. The recovered cassava plants with the non-persistent SLCMV, which were maintained vegetative in quarantine in the University of Basel (Basel, Switzerland) greenhouse, displayed re-emergence of CMD after a six-month period. Interestingly, these plants did not carry SLCMV but carried ICMV. It is interpreted that the field-collected, SLCMV-infected cassava plants were co-infected with low levels of ICMV. The loss of SLCMV in recovered cassava plants, under greenhouse conditions, then facilitated the re-emergence of ICMV. The partial dimer clones of the persistent and non-persistent isolates of SLCMV and the re-emerged isolate of ICMV were infective in *Nicotiana benthamiana* upon agroinoculation. Studies on pseudo-recombination between SLCMV and ICMV in *N. benthamiana* provided evidence for trans-replication of ICMV DNA B by SLCMV DNA A.

## 1. Introduction

Geminiviruses are circular single-stranded DNA (ssDNA) viruses of the family *Geminiviridae* that are encapsidated in paired icosahedral particles [1]. Geminiviruses are classified on the basis of their genome organization and vector transmission into seven genera: *Begomovirus*, *Mastrevirus*, *Curtovirus*, *Becurtovirus*, *Eragrovirus*, *Topocuvirus* and *Turncurtovirus* [2,3]. Begomoviruses infect mostly dicotyledonous plants and are transmitted by the whitefly *Bemisia tabaci* [4].

Cassava (*Manihot esculenta* Crantz, Family Euphorbiaceae) is a tropical food crop and one of the major commercial crops of Africa, the Indian sub-continent, Latin America and several Southeast Asian countries. Cassava mosaic disease (CMD), caused by cassava mosaic geminiviruses (CMGs), is the main constraint for cassava cultivation in Africa and Indian sub-continent [5,6]. In India, cassava is mainly grown in Kerala, Tamil Nadu and Andhra Pradesh states.CMD was first reported in India by Abraham [7] and later studied in detail by Alagianagalingam and Ramakrishnan [8]. CMGs are bipartite begomoviruses and the genome is organized into DNA A and DNA B components. DNA A encompasses two genes (*AV1* and *AV2*) in the virion-sense strand and four genes (*AC1*, *AC2*, *AC3* and *AC4*) in the complementary-sense strand. The genes in DNA A encode proteins involved in replication, transcriptional activation, encapsidation and silencing suppression.DNA B encompasses two genes (*BV1* and *BC1*), which assist movement of the virus from the nucleus to the cytoplasm and also from one cell to the other [9]. Indian cassava mosaic virus (ICMV) and Sri Lankan cassava mosaic virus (SLCMV) cause CMD in India [9,10,11]. Both ICMV and SLCMV also infect *Nicotiana* spp. SLCMV in particular is highly virulent with a broad host-range extending to *Arabidopsis* [12].

Geminiviruses trigger gene silencing which enables the host plants to recover from viral infection [13,14]. Recovery or symptom remission was observed in many geminivirus-infected plants. One such recovery pattern was studied in *Capsicum annum*, which recovered from the infection of Pepper golden mosaic virus (PepGMV) [15,16].

Gene silencing and recovery from CMGs are isolate-dependent [13]. African cassava mosaic virus (ACMV) and SLCMV infections result in higher levels of small interfering RNA (siRNA) accumulation in the recovered plants, whereas isolates that caused non-recovering infection lead to accumulation of lower levels of siRNAs.A correlation between siRNA accumulation and reduction in viral titer was reported in some cucurbits, which showed recovery from Cucurbit leaf crumple virus (CuLCrV) [17]. Elevated temperature, which causes an increase in the siRNA accumulation in plants, also induces recovery of plants from CMD [18,19].

Trans-replication and pseudo-recombination can occur between two different species of begomoviruses in co-infected plants. Pseudo-recombination was reported between Tomato mottle virus (ToMoV) and Bean dwarf mosaic virus (BDMV) [20]. Subsequently, pseudo-recombination between other bipartite begomoviruses has been documented [21,22]. Replication associated protein (Rep) is a multifunctional protein involved mainly in the replication of geminiviral DNA by rolling circle replication (RCR) [23]. Iterative sequences or iterons are the Rep recognition sites present in the common region (CR) of geminiviral DNA A and DNA B, which determine the specificity of Rep binding and facilitate the replication of cognate DNA A and DNA B molecules [24]. ACMV and SLCMV have identical iterons, which can produce infectious pseudo-recombinants, whereas ICMV differs in the iteron sequences, resulting in replicational incompatibility with both ACMV and SLCMV [9].

In our current study, we came across a persistent and a non-persistent SLCMV isolate, infecting the same Sengutchi cultivar of cassava in the cassava fields near the cities Malappuram and Thiruvananthapuram. Cassava plants, which initially recovered from the non-persistent SLCMV infection, later displayed CMD symptoms again in the greenhouse. The symptomatic plants intriguingly carried ICMV DNA A and DNA B. DNA A and DNA B partial dimers of the two SLCMV isolates and the ICMV isolate caused viral disease upon agroinoculation of *Nicotiana benthamiana*. The agroinoculation studies further showed that SLCMV DNA A could trans-replicate ICMV DNA B. This is the first report of pseudo-recombination between SLCMV and ICMV.

## 2. Material and Methods

### 2.1. Viral Clones

Field-collected cassava plants (*Manihot esculenta* Crantz, cultivar Sengutchi) collected from Malappuram and Thiruvananthapuram in June 2009 were propagated as stem cuttings initially in the Madurai Kamaraj University (MKU) greenhouse under natural light (12 h light/12 h darkness, 23 to 33 °C). Details of SLCMV and ICMV isolates, their National Center for Biotechnology Information (NCBI) accession numbers, the sizes of their DNA A and DNA B components and the isolates to which the maximum nucleotide sequence identity is seen are presented in Table 1. SLCMV-Sengutchi Malappuram [SeM] DNA A was cloned by polymerase chain reaction (PCR) using abutting primers. SLCMV-[SeM] DNA B, SLCMV-Sengutchi Thiruvananthapuram [SeT1] DNA A, SLCMV-[SeT1] DNA B, ICMV-[SeT4] DNA A and ICMV-[SeT4] DNA B were cloned by rolling circle amplification (RCA) [25] using *phi*29 DNA polymerase (GE Healthcare UK Ltd., Little Chalfont, UK).

### 2.2. Diagnostic Multiplex PCR Analysis for Amplification of SLCMV and ICMV

Diagnostic multiplex PCR for amplification of SLCMV and ICMV was carried out by using primers which specifically amplify SLCMV or ICMV DNA A fragments. SLCMV-specific primers 5′-GAAGGGAGACACATATACCTCG-3′ and 5′-CACATATATATTGTCTCCAATTCAC-3′ were used for the amplification of a 615 bp SLCMV DNA A fragment. ICMV-specific primers 5′-AGAAAGGGTTTTGATACGGAG-3′ and 5′-CTCATCTCCACGTGCTCATC-3′ were used for the amplification of a 386 bp ICMV DNA A fragment.

### 2.3. Construction of SLMV-[SeM] DNA A and DNA B Partial Dimers

SLCMV-[SeM] DNA A (2756 bp) was cloned as a PstI fragment in pBSIIKS^+^ to yield pBS-SLCMV-Ma-A. A 1.7 kb PstI/HindIII fragment of DNA A from pBS-SLCMV-Ma-A (0.6-mer with CR) was cloned into the corresponding sites of the binary vector pPZP201 [26] to yield pPZP-SLCMV-Ma0.6A. The 2.7 kb full-length DNA A from pBS-SLCMV-Ma-A was taken as a PstI fragment (1-mer) and cloned in the PstI site of pPZP-SLCMV-Ma0.6A to yield the partial dimer binary plasmid pPZP-SLCMV-Ma1.6A.

SLCMV-[SeM] DNA B (2737 bp) was cloned as a BamHI fragment in pBSIIKS^+^ to yield pBS-SLCMV-Ma-B.A 2.0 kb BamHI/HindIII fragment of DNA B (0.7-mer with CR) from pBS-SLCMV-Ma-B was cloned in the corresponding sites of pBSIIKS^+^ to yield pBS-SLCMV-Ma0.7B. The 2.7 kb full-length DNA B from pBS-SLCMV-Ma-B was taken as a BamHI fragment (1-mer) and cloned in the BamHI site of pBS-SLCMV-Ma0.7B to yield the partial dimer plasmid pBS-SLCMV-Ma1.7B. A 4.7 kb SacI/SalI fragment which comprises the partial dimer was cloned in the binary vector pPZP201 to yield pPZP-SLCMV-Ma1.7B.

### 2.4. Construction of SLCMV-[SeT1] DNA A and DNA B Partial Dimers

SLCMV-[SeT1] DNA A (2746 bp) was cloned as a PstI fragment in pBSIIKS^+^ to yield pBS-SLCMV-Tv-A. A 1.7 kb PstI/HindIII fragment of DNA A (0.6-mer with CR) from pBS-SLCMV-Tv-A was cloned in the corresponding sites of pPZP201 to yield the plasmid pPZP-SLCMV-Tv0.6A. The 2.7 kb full-length DNA A from pBS-SLCMV-Tv-A was cloned as a PstI fragment (1-mer) in the PstI site of pPZP-SLCMV-Tv0.6A to yield the partial dimer binary plasmid pPZP-SLCMV-Tv1.6A.

SLCMV-[SeT1] DNA B (2739 bp) was cloned as a BamHI fragment in pBSIIKS^+^ to yield pBS-SLCMV-Tv-B. A 1.7 kb BamHI/BglII fragment (0.6-mer with CR) of DNA B from pBS-SLCMV-Tv-B was cloned in the BamHI site of pPZP201 to yield pPZP-SLCMV-Tv0.6B. The 2.7 kb full-length DNA B (1-mer) was taken from pBS-SLCMV-Tv-B as a BamHI fragment and cloned in the BamHI site of pPZP-SLCMV-Tv0.6B to yield the partial dimer binary plasmid pPZP-SLCMV-Tv1.6B.

### 2.5. Construction of ICMV-[SeT4] DNA A and DNA B Partial Dimers

ICMV-[SeT4] DNA A (2735 bp) was cloned in pBSIIKS^+^ as a PstI fragment to yield pBS-ICMV-Tv-A. A 2.3 kb XbaI/PstI fragment (0.8-mer with CR) from pBS-ICMV-Tv-A was cloned in the corresponding sites of pBSIIKS^+^ to yield pBS-ICMV-Tv0.8A. The 2.7 kb full-length DNA A was taken from pBS-ICMV-Tv-A as a PstI fragment (1-mer) and cloned in the PstI site of pBS-ICMV-Tv0.8A to yield the partial dimer pBS-ICMV-Tv1.8A. The 5.0 kb partial dimer was then taken as a SacI/SalI fragment and cloned in the corresponding sites of pPZP201 to yield the partial dimer binary plasmid pPZP-ICMV-Tv1.8A.

ICMV-[SeT4] DNA B (2716 bp) was cloned as a BamHI fragment in pBSIIKS^+^ to yield pBS-ICMV-Tv-B. A 1.7 kb BamHI/BglII fragment (0.6-mer with CR) from pBS-ICMV-Tv-B was cloned in the BamHI site of pPZP201 to yield pPZP-ICMV-Tv0.6B. The full-length DNA B (1-mer) was taken as a 2.7 kb BamHI fragment from pBS-ICMV-Tv-B and cloned in the BamHI site of pPZP-ICMV-Tv0.6B to yield the partial dimer binary plasmid pPZP-ICMV-Tv1.6B.

All binary plasmids with partial dimers were independently mobilized into the *Agrobacterium tumefaciens* strain Ach5 by triparental mating [27] and the mobilizations were confirmed by Southern blot analysis.

### 2.6. Agroinfection of *Nicotiana benthamiana*

Three-week-old *N. benthamiana* plants were agroinoculated with the *A. tumefaciens* Ach5 strains harbouring the DNA A and DNA B partial dimers by the two strain method [28]. *A. tumefaciens* Ach5 strain without partial dimers (for mock inoculation) and with partial dimers were grown in AB minimal medium [29] to optical density at 600 nm = 1 and the cells were centrifuged (1100× *g*, Hitachi himac CR 20B2, Rotor 7, Hitachi Koki Co., Ltd., Tokyo, Japan) at 28 °C. The pellets were re-suspended in AB minimal medium (pH 5.6) with 100 μM acetosyringone (Sigma-Aldrich, St. Louis, MO, USA). The cultures were either taken separately for infection or mixed in 1:1 ratio for co-infection of DNA A + DNA B. The stem of *N. benthamiana* plants was pricked three times with a 30G needle above the first fully expanded young leaf and agroinoculation was performed by inoculating 10 μL of the re-suspended *Agrobacterium* cultures at the pricked area [30,31].

### 2.7. Southern Blot Analysis

Total DNA from cassava was extracted using the Nucleon Phytopure Genomic DNA Extraction Kit (GE Healthcare UK Ltd.). DNA from *N. benthamiana* was extracted as described by Rogers and Bendich [32]. DNA was estimated in a fluorimeter (DyNA Quant 200, Hoefer Scientific Instruments, San Francisco, CA, USA) using the Hoechst dye 33258 (Polysciences Inc., Warrington, PA, USA). One microgram plant DNA samples were electrophoresed in 0.8% agarose gels in 1X Tris-sodium acetate-ethylenediaminetetraacetic acid (EDTA) (TNE) buffer [33]. Following ethidium bromide staining and alkali denaturation, DNA was transferred [34] to the Zetaprobe nylon membrane (Bio-Rad Laboratories, Hercules, CA, USA). The probe DNA was labeled with [α-^32^P] deoxycytidine triphosphate (dCTP) using the Megaprime DNA labeling system (GE Healthcare UK Ltd.). Hybridization was carried out overnight at 65 °C followed by high stringency post hybridization washes [35].

### 2.8. siRNA Analysis

Total RNA from cassava plants was extracted using the Tri Reagent (Sigma-Aldrich, St. Louis, MO, USA). SpeedVac-concentrated total RNA (10 μg) was electrophoresed in a 15% polyacrylamide gel with 8 M urea at 300 V. The separated RNA was blotted onto the nylon membrane (Roche Diagnostics, Indianapolis, IN, USA) in 1X Tris/Borate/EDTA buffer (TBE) using Transblot-SD semidry transfer apparatus (Bio-Rad Laboratories) at 7 V for 45 min. After blotting, the membrane was UV cross-linked twice. A 1.0 kb SLCMV *AC1* fragment was labeled with [α-^32^P]dCTP using the Megaprime DNA labeling system and used as the probe. Hybridization was carried out for 16–20 h at 37 °C followed by post-hybridization washes for four times with 2X saline sodium citrate (SSC)/0.2% sodium dodecyl sulfate (SDS) for 20 min each at 50 °C [36].

### 2.9. Illumina Sequencing and Bioinformatic Analysis of Small RNAs

**Figure 1:**
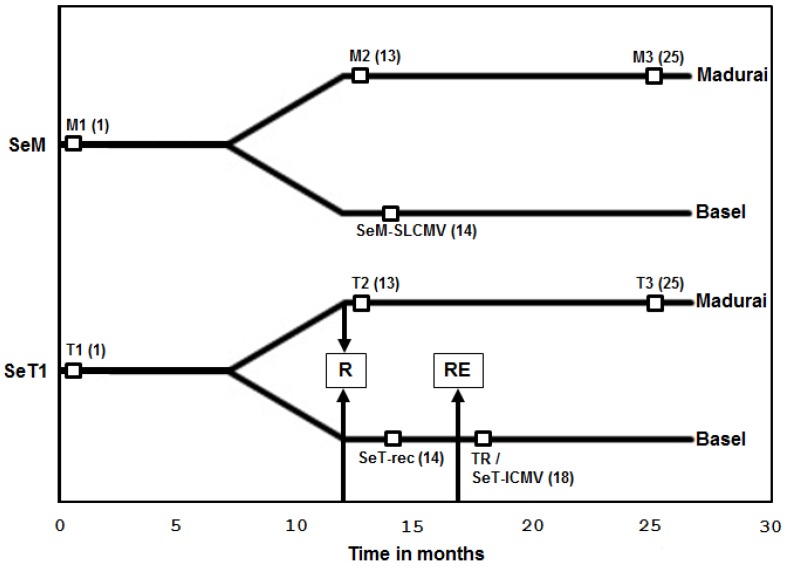
Timelines of cassava plant establishment in Madurai and Basel greenhouses and periods at which samples were taken for DNA and RNA extraction. The periods (in months) following the initial establishment of cassava plants (*Manihot esculenta* Crantz) in Madurai are represented in the *x*-axis. SeM, the cassava plant collected from Malappuram; SeT1, the cassava plant collected from Thiruvananthapuram. The time point 0 indicates the time of initial establishment of field-infected cassava plants in the Madurai greenhouse. The plants were established in the Basel greenhouse eight months after initial establishment in Madurai. R denotes the time of recovery of the SeT 1 plant from cassava mosaic disease (CMD) and RE denotes the time of re-emergence of CMD symptoms. M1, M2 and M3 are the time points at which DNA was extracted from SeM plants. T1, T2 and T3 are the time points at which DNA was extracted from SeT1 plants (both maintained in the Madurai greenhouse). TR is the time point of re-emergence of CMD in SeT1 cassava plants grown in the Basel greenhouse. Time points of RNA extraction in SeM plants (SeM-SLCMV), SeT1 plants at the recovery stage (SeT-rec) and SeT plants at the re-emergent stage (SeT-ICMV) are marked. The number of months from the initial establishment of cassava plants in Madurai is given in brackets.

**Figure 2:**
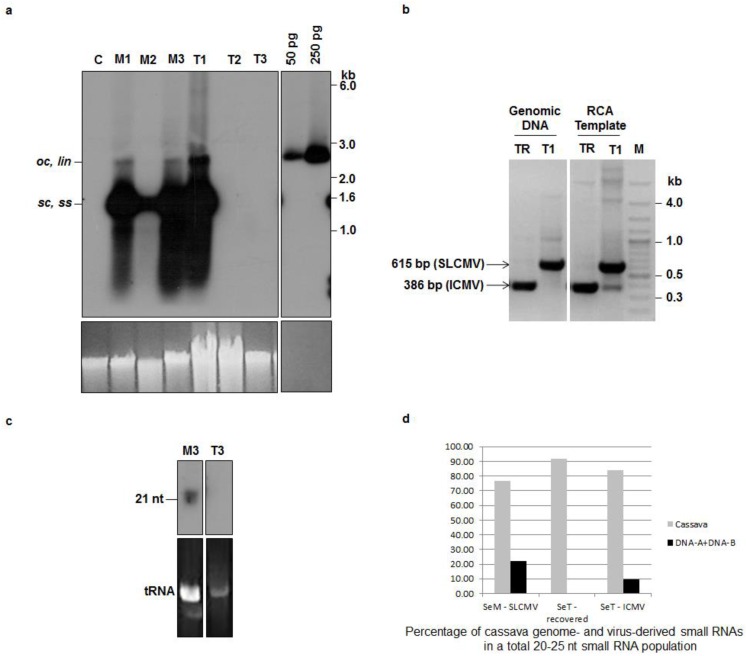
Sri Lankan cassava mosaic virus (SLCMV) DNA A and small interfering RNA (siRNA) analysis in symptomatic and symptom-recovered cassava plants. (**a**) Southern blot analysis of field-infected cassava plants maintained in the greenhouse. DNA (1 μg) samples from a virus-free, axenic (tissue culture-grown) cassava plant (C), and field-infected cassava (cultivar Sengutchi) plants collected from Malappuram (M) and Thiruvananthapuram (T) were analyzed. The field-infected cassava plants were established in the Madurai Kamaraj University (MKU) greenhouse and the DNA was extracted during first year (M1 and T1), second year (M2 and T2) and third year (M3 and T3) of establishment in the greenhouse (Figure 1). pBS-SLCMV-Ma-A plasmid (50 pg and 250 pg) digested with PstI was used as a positive control. [α-^32^P]dCTP-labeled full-length SLCMV-[SeM] DNA A was used as the probe. Positions of different forms of viral DNA, single stranded (*ss*), super-coiled (*sc*), open circular (*oc*) and linear (*lin*), are marked. Ethidium bromide stained high molecular weight plant DNA is shown as loading control at the bottom panel. (**b**) Diagnostic multiplex PCR using SLCMV- and ICMV-specific primers to analyze mixed infection in the plant sample T1 (field-infected SLCMV-SeT1, symptomatic plant) and TR (plant which initially recovered and subsequently showed re-emergence of CMD symptoms in the Basel greenhouse) (Figure 1). Genomic DNA and RCA-amplified DNA from T1 and TR were used as templates for polymerase chain reaction (PCR) analysis. Amplified fragments of SLCMV (615bp) and Indian cassava mosaic virus (ICMV) (386bp) are marked. M, Molecular weight marker. (**c**) Small RNA Northern blot analysis of field-infected cassava plants. RNA (10 μg) from field-infected cassava plants collected from Malappuram (M3) and Thiruvananthapuram (T3) were analyzed. The field-infected cassava plants were established in the MKU greenhouse and RNA was extracted after 25 months of initial establishment in the greenhouse (M3 and T3). [α-^32^P]dCTP-labeled SLCMV-[SeM] *AC1* (1 kb) gene fragment was used as the probe. The position of siRNA (21 nt) is marked. The bottom panel shows ethidium bromide stained tRNA, as a loading control. (**d**) The proportion of the plant genome (cassava)- and the viral genome (DNA A + DNA B)-derived small RNAs in a total population of 20–25nt small RNAs accumulating in the Sengutchi cassava plants “SeM-SLCMV”, “SeT-recovered” and “SeT-ICMV” (Figure 1).

Total RNA was isolated from upper leaves of three Sengutchi cassava plants (Figure 1): (i) “SeM-SLCMV”, the symptomatic plant from Malappuram which was persistently infected in Basel with SLCMV-[SeM]); (ii) “SeT-recovered”, the symptomless plant from Thiruvananthapuram which had recovered in Basel from infection with SLCMV-[SeT1]; and (iii) “SeT-ICMV”, a vegetative progeny of the “SeT-recovered” plant which eventually exhibited disease symptoms and found to be infected with re-emergent ICMV-[SeT4] (see below).cDNA libraries were prepared from 19 to 30 ntRNA fractions following an Illumina Small RNA TruSeq protocol and sequenced on the Illumina Genome Analyzer HiSeq2000 using a TruSeq SBS Kit v3 at Fasteris AG [37]. After trimming the adaptor sequences, 20–25 nt small RNA (sRNA) reads were mapped to the draft genome of *Manihot esculenta* v6.1 [38] and the genome of the respective virus present in each plant (i.e., SLCMV-[SeM], SLCMV-[SeT1], or ICMV-[SeT4]) using the Burrows-Wheeler Alignment Tool (BWA, Version 0.5.9) [39] with up to two mismatches. Percentage of the plant and the viral reads in the total population of 20–25 nt reads was calculated and presented as a bar graph in Figure 2d.

## 3. Results and Discussion

### 3.1. Recovery of Cassava Plants from SLCMV Infection in the Greenhouse and Re-Emergence of Symptoms due to Latent ICMV

The vegetative progeny of the infected cassava plants were transferred from MKU as stem-cuttings and established in the greenhouse of the University of Basel in February 2010. The timelines of cassava plant establishment in the Madurai and Basel greenhouses and periods of sample collection are illustrated in Figure 1. DNA was extracted from the two plants (M1 and T1) at the initial stage of greenhouse establishment in MKU and the CMD was confirmed by PCR and Southern blot analysis (data not shown). The full-length viral DNA A was amplified from the plant samples M1 and T1 by either PCR or RCA. The amplified fragments of 2.7 kb were cloned and sequenced. Both Sengutchi plants were found to be infected with SLCMV (Table 1).

The Sengutchi plant collected from Malappuram (infected with SLCMV-[SeM]) showed persistence of CMD symptoms throughout the study period of three years in the MKU greenhouse. However, the Sengutchi plants collected from Thiruvananthapuram (infected with SLCMV-[SeT1]) and maintained in the same facility showed recovery from the CMD symptoms from the second year. Southern blot analysis using a SLCMV DNA A probe in the second (T2) and third years (T3) revealed the absence of viral DNA in the cassava plants originally infected with the SLCMV-[SeT1] isolate (Figure 2a). This shows that SLCMV-[SeT1] is a non-persistent virus isolate. Cassava plants with SLCMV-[SeM] infection maintained viral titer in all the three samples M1, M2 and M3, collected at the beginning of 1st, 2nd and 3rd years, respectively (Figure 2a). Thus, SLCMV-[SeM] is a persistent virus isolate.

The Sengutchi cassava plants, from which the SLCMV-[SeT1] was cloned, displayed initially CMD symptoms in the Basel greenhouse. Recovery from the CMD symptoms and absence of SLCMV was observed in the plants established in both the Basel and Madurai greenhouses. Interestingly, the CMD symptoms re-emerged in those plants during October 2010, after eight months of initial establishment at Basel. DNA was extracted from those plants, the viral DNA was cloned by RCA and the full-length viral DNA A (2735 nt) and DNA B (2716 nt) clones were sequenced (Table 1). The sequences revealed that ICMV was responsible for re-emergence of CMD symptoms.The Basel greenhouse did not contain any other ICMV-infected plants. Cassava plants are not grown in Basel and their viruses are absent. Therefore, the presence of ICMV DNA A and DNA B in the re-emergent infection of cassava plants should be attributed to the re-emergence of ICMV from a latent infection, which was undetectable by PCR at the time of field collection. We therefore carried out an additional experiment to detect the latent ICMV in the original DNA sample of plant T1 (Figure 1) extracted from the Sentgutchi plant (SeT1) at Madurai. Using RCA amplification of circular viral DNA followed by a diagnostic multiplex PCR analysis with the ICMV DNA A and the SLCMV DNA A specific primers, we detected in fact a very low level of ICMV DNA A in addition to abundant SLCMV DNA A in the plant T1 (Figure 2b).

Virus induced post-transcriptional gene silencing (PTGS) in cassava results in recovery from ACMV and SLCMV symptoms, which correlates well with the accumulation of siRNAs [13]. Analysis of viral siRNAs was performed in Sengutchi cassava plants infected with persistent and non-persistent SLCMV isolates. *AC1* was used as the probe, since we detected a high level of siRNAs in field-infected cassava plants with this probe. The Sengutchi cassava plant M3 (Figure 1), which was infected with the persistent isolate SLCMV-[SeM], showed accumulation of siRNAs corresponding to the *AC1* gene (Figure 2c). However, the plant T3 (Figure 1), which was originally infected with the non-persistent isolate SLCMV-[SeT1] and later showed recovery from CMD, did not accumulate virus-derived siRNAs (Figure 2c). The recovery from CMD was expected because of strong silencing (more viral siRNAs). However, siRNA accumulation was not observed in the plant T3. The time point T3 (Figure 1, 25 months) is 13 months past the recovery stage (12 months) and also the insufficient loading of T3 could have resulted in the absence of siRNAs detection. Therefore, a second set of recovered SeT1 samples at the 14-month time point was taken for deep sequencing of small RNAs. We deep-sequenced small RNAs from the vegetative progeny of the Sentgutchi-Thiruvananthapuram plant that had recovered from CMD caused by SLCMV-[SeT1] (designated “SeT-rec”) (Figure 1) and later displayed CMD in Basel owing to re-emergence of the latent ICMV (designated “SeT-ICMV”) (Figure 1), as well as from the vegetative progeny of the Sentgutchi-Malappuram plant showing persistent CMD (caused by SLCMV-[SeM]; designated “SeM-SLCMV”) (Figure 1). Bioinformatics analysis of the deep sequencing data revealed that both symptomatic plants accumulated high proportion of viral siRNAs in a total population of 20–25 nt sRNAs (9.7% in the SeT-ICMV plant and 22% in the SeM-SLCMV plant) (Figure 2d). In contrast, only trace amounts of viral siRNAs were detected in the SeT-rec plant (0.07% of the total 20–25 nt sRNAs). Taken together, our findings agree with the findings of Patil and Fauquet [19], which showed that recovery from symptoms is not always associated with high siRNA accumulation. The re-emergence of CMD symptoms from recovered plants occurred after the complete loss of the SLCMV-[SeT1] titer and near complete loss of viral siRNAs, and this re-emergence of severe disease symptoms was associated with production of viral siRNAs from ICMV-[SeT4] that replicated efficiently in the absence of SLCMV. SLCMV is more widespread in the field in contrast to ICMV in the Indian sub-continent [40] possibly because ICMV does not replicate efficiently in the presence of SLCMV. An alternative hypothesis is that the presence of ICMV in the SeT1 plants caused recovery, whereas the absence of ICMV in the SeM plants did not lead to recovery.

### 3.2. Infectivity of Cloned SLCMV DNA A and DNA B in *Nicotiana benthamiana* Plants

**Figure 3:**
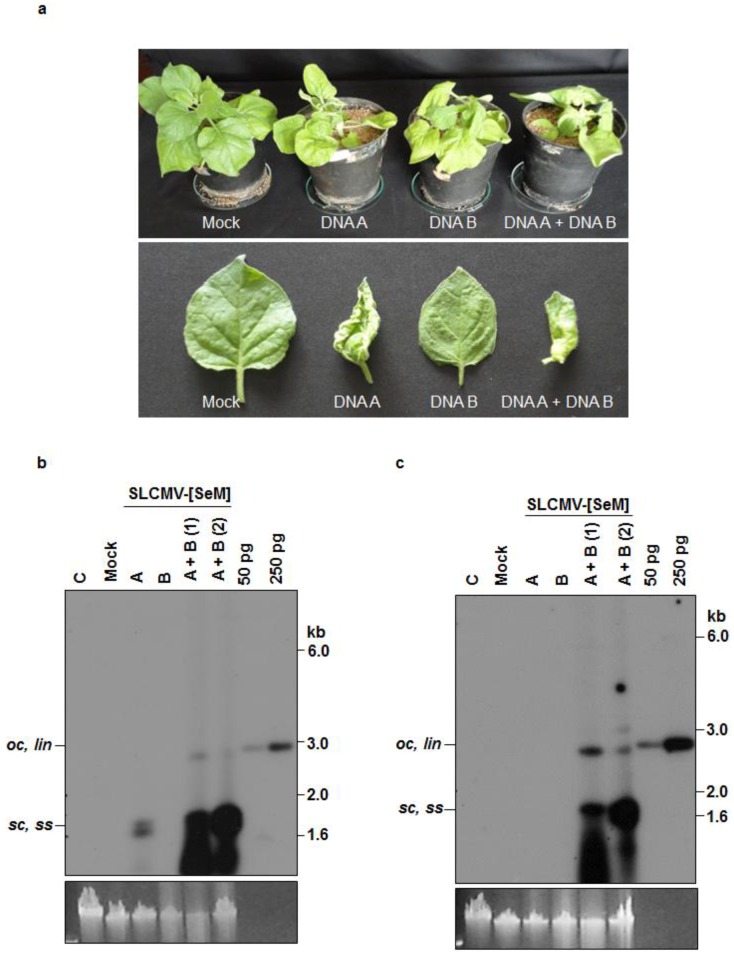
Infectivity analysis of SLCMV-[SeM] partial dimers in *N. benthamiana* plants. (**a**) Symptoms displayed by *N. benthamiana* plants agroinoculated with the SLCMV-[SeM] partial dimers. Bottom half shows individual leaves of the tested plants. (**b**) Southern blot analysis using SLCMV-[SeM] DNA A (without common region, ΔCR) labeled with [α-^32^P]dCTP as the probe. The plasmid pBS-SLCMV-Ma-A digested with PstI (50 pg and 250 pg) was used as the positive control. (**c**) Southern blot analysis using [α-^32^P]dCTP-labeled SLCMV-[SeM] DNA B (ΔCR) as the probe. The plasmid pBS-SLCMV-Ma-B digested with BamHI (50 pg and 250 pg) was used as the positive control. (**b**,**c**) DNA (1 μg) from uninfected plant (C), plant mock infected with the *Agrobacterium tumefaciens* strain Ach5 (Mock), plant agroinoculated with partial dimers of DNA A alone (A), DNA B alone (B) and two plants independently co-agroinoculated with the partial dimers of DNA A + DNA B (A + B) were loaded in the respective lanes. Positions of different forms of viral DNA, single stranded (*ss*), super-coiled (*sc*), open circular (*oc*) and linear (*lin*), are marked. Ethidium bromide stained high molecular weight plant DNA is shown as loading control at the bottom.

The infectivity of partial dimers of cloned DNA A and DNA B components of SLCMV-[SeM] and SLCMV-[SeT1] were analyzed in three-week-old *N. benthamiana* plants by agroinfection (Figure 3 and Figure S1). Plants were scored for symptoms 14-day-post infection. For both SLCMV isolates, plants agroinoculated with DNA A showed upward leaf rolling symptom, while the plants agroinoculated with DNA B remained symptomless (Table 2). Plants co-agroinoculated with both SLCMV DNAs (A and B) showed more severe typical geminiviral symptoms of stunting, chlorosis and downward leaf curling in all the agroinoculated plants (Figure 3a and Figure S1a). For both SLCMV isolates, Southern blotting revealed much higher DNA A levels in plants agroinoculated with DNA A and DNA B than with DNA A alone (Figure 3b and Figure S1b). Plants agroinoculated with DNA B alone, as expected, did not accumulate the viral DNA. Using a probe lacking the common region (ΔCR), DNA B accumulation was observed only in plants co-agroinoculated with DNA A + DNA B (Figure 3c and Figure S1c).

The upward leaf rolling symptom observed upon agroinoculation with DNA A alone is typical for monopartite geminiviruses, suggesting the evolution of SLCMV from a monopartite geminivirus by the acquisition of DNA B from ICMV [9]. Infectivity analysis of SLCMV-[SeM] and SLCMV-[SeT1] in *N. benthamiana* plants proved that the clones of both isolates from field-infected cassava plants (cultivar Sengutchi) are infectious.

### 3.3. Infectivity of Cloned ICMV DNA A and DNA B in *Nicotiana benthamiana* Plants

**Figure 4:**
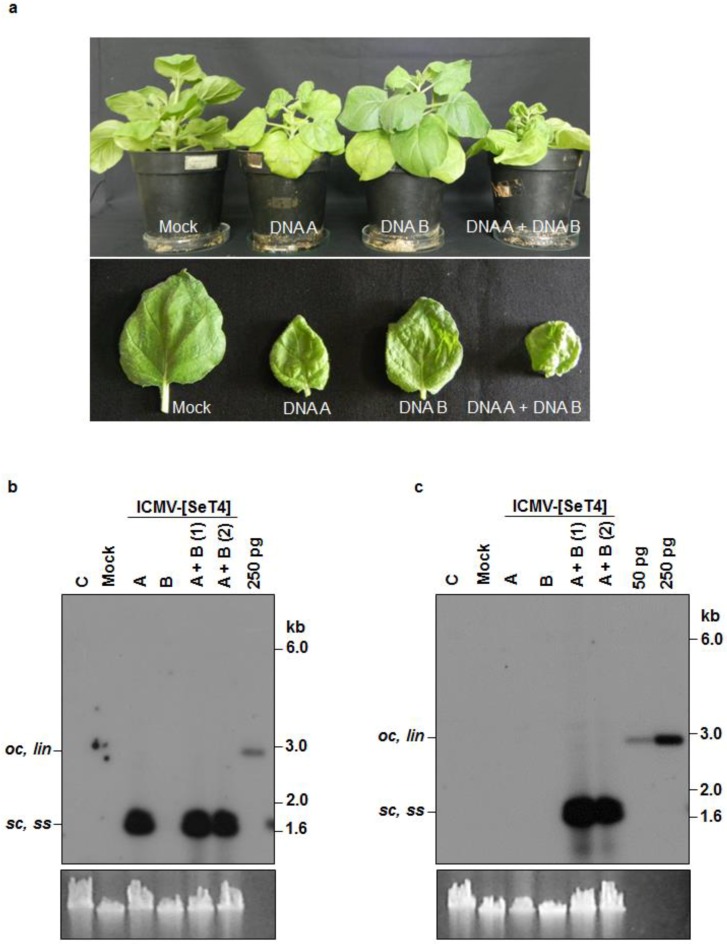
Infectivity analysis of ICMV-[SeT4] in *N. benthamiana* plants. (**a**) Symptoms displayed by *N. benthamiana* plants agroinoculated with the partial dimers of ICMV-[SeT4]. Bottom half shows individual leaves of the tested plants. (**b**) Southern blot analysis using ICMV-[SeT4] DNA A (ΔCR) labeled with [α-^32^P]dCTP as the probe. The plasmid pBS-ICMV-Tv-A digested with PstI (250 pg) was used as the positive control. (**c**) Southern blot analysis using [α-^32^P]dCTP-labeled ICMV-[SeT4] DNA B (ΔCR) as the probe. The plasmid pBS-ICMV-Tv-B digested with BamHI (50 pg and 250 pg) was used as the positive control. (**b**,**c**) DNA (1 μg) from uninfected plant (C), plant mock infected with the *A. tumefaciens* strain Ach5 (Mock), plant agroinoculated with the partial dimers of DNA A alone (A), DNA B alone (B) and two plants independently co-agroinoculated with the partial dimers of DNA A + DNA B (A + B) were loaded in the respective lanes. Positions of different forms of viral DNA, single stranded (*ss*), super-coiled (*sc*), open circular (*oc*) and linear (*lin*), are marked. Ethidium bromide stained high molecular weight plant DNA is shown as loading control at the bottom.

Similar agroinfection experiments were performed with ICMV partial dimers (Table 2). All plants agroinoculated with ICMV-[TVM4] DNA A alone showed very mild leaf blade curling symptoms (Figure 4a). Interestingly, upward leaf rolling as noted for SLCMV DNA A agroinoculation was not observed. All plants agroinoculated with DNA A + DNA B displayed more severe symptoms (i.e., stunting and leaf blade curling). Again, none of the plants agroinoculated with DNA B alone displayed symptoms.

In the ICMV case and in contrast to the SLCMV case, Southern blotting revealed comparable levels of DNA A accumulation in the presence and absence of DNA B (Figure 4b). No DNA B accumulation was observed in plants agroinoculated with the DNA B alone (Figure 4c).

The fact that ICMV DNA A accumulated to high levels, regardless whether DNA B was present or not, while symptoms were much more severe upon agroinoculation with both DNAs, revealed that ICMV DNA B plays an important role in symptom development, which validates the earlier reports on symptom determination of bipartite begomoviruses [31,35,41]. The infectivity analysis of ICMV-[SeT4] in *N. benthamiana* plants proved that the isolate cloned from the CMD re-emergent cassava plants is infectious.

### 3.4. Analysis of Trans-Replication of ICMV-[TVM4] DNA B by SLCMV-[SeM] DNA A

**Figure 5:**
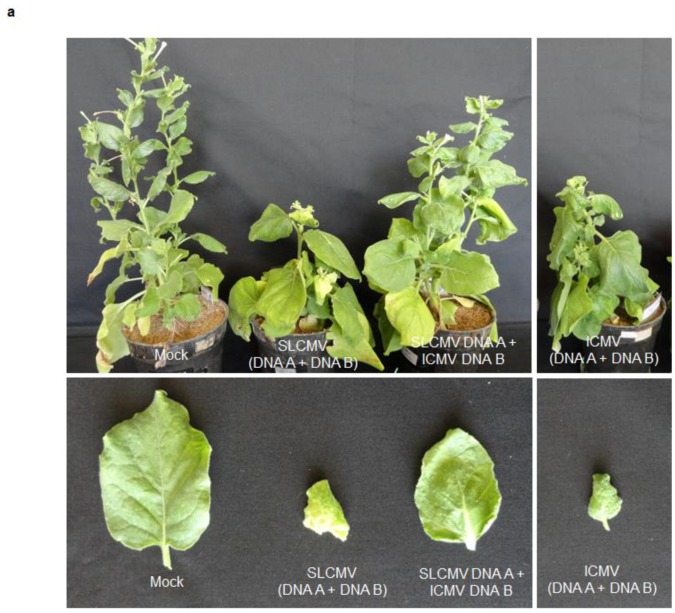

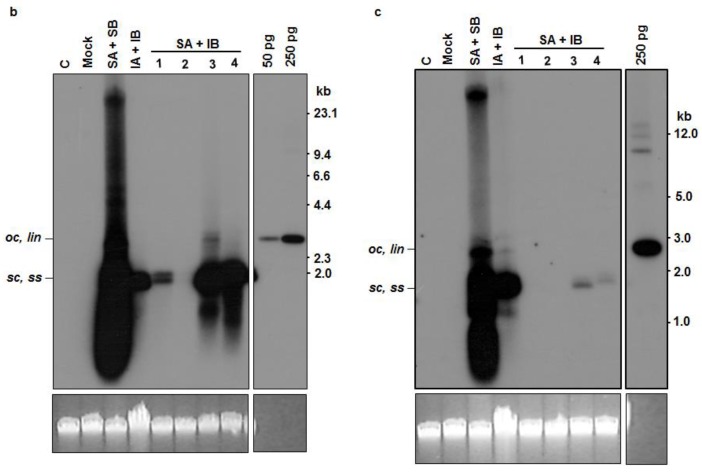
Pseudo-recombination in *N. benthamiana* with SLCMV-[SeM] DNA A and ICMV-[SeT4] DNA B. (**a**) Symptoms in *N. benthamiana* mock inoculated plants (Mock) and plants agroinoculated with the partial dimers of SLCMV-[SeM] DNA A + DNA B, ICMV-[SeT4] DNA A + DNA B and SLCMV-[SeM] DNA A + ICMV-[SeT4] DNA B. (**b**) Southern blot analysis using SLCMV-[SeM] DNA A (ΔCR) labeled with [α-^32^P]dCTP as the probe. The plasmid pBS-SLCMV-Ma-A digested with PstI (50 pg and 250 pg) was used as the positive control. (**c**) Southern blot analysis using [α-^32^P]dCTP-labeled SLCMV-[SeM] DNA B (ΔCR) as the probe. The plasmid pBS-SLCMV-Ma-B digested with BamHI (250 pg) was used as the positive control. (**b**,**c**) DNA (1 μg) from uninfected *N. benthamiana* plant (C), plant mock infected with the *A. tumefaciens* strain Ach5 (Mock), plant co-agroinoculated with the partial dimers of SLCMV-[SeM] DNA A + DNA B (SA + SB), plant co-agroinoculated with the partial dimers of ICMV-[SeT4] DNA A + DNA B (IA + IB) and plants co-agroinoculated with the partial dimers of SLCMV-[SeM] DNA A + ICMV-[SeT4] DNA B (SA + IB) were loaded in the respective lanes. Positions of different forms of viral DNA, single stranded (*ss*), super-coiled (*sc*), open circular (*oc*) and linear (*lin*), are marked. Ethidium bromide stained high molecular weight plant DNA is shown as loading control at the bottom.

SLCMV-[SeM] DNA A and ICMV-[SeT4] DNA B partial dimers were used to study pseudo-recombination. The infectivity rate in plants co-agroinoculated with SLCMV DNA A + ICMV DNA B was lower than in plants co-agroinocculated with either the SLCMV or ICMV cognate pairs. Two of the four SLCMV DNA A + ICMV DNA B co-agroinoculated plants displayed mild leaf blade curling (Figure 5a) and showed high levels of SLCMV DNA A and low levels of DNA B (Figure 5b,c, lanes 3 and 4).

The leaf blade curling symptom of SLCMV-A/ICMV-B may be associated with the presence of ICMV DNA B, which had elevated the level of SLCMV DNA A accumulation (Figure 5b,c, lanes 3 and 4). The result suggests that SLCMV-[SeM] DNA A trans-replicates ICMV DNA B, while systemic viral movement mediated by ICMV DNA B may be responsible for higher SLCMV DNA A accumulation and enhanced symptoms in comparison to SLCMV DNA A alone infected plants (data not shown). Trans-replication elevated the viral DNA A level possibly by elevating viral movement by DNA B.

Pseudo-recombination was earlier reported between ACMV and SLCMV, which share an identical iteron sequence (5′ AATTGGAGACA 3′) [9]. In the present pseudo-recombination study, ICMV DNA B, which has a non-identical iteron sequence (5′ GGTACTCA 3′) in comparison to that of SLCMV DNA A (5′ AATTGGAGACA 3′), also showed trans-replication. The accumulation of a low level of ICMV DNA B, increased the level of SLCMV DNA A and had an impact on the development of symptoms.

The Rep binding motif (5′ AATCGGTGTC 3′) of the Tomato leaf curl virus (TLCV) is present in the TLCV satellite DNA (sat-DNA) and the replication of this sat-DNA is supported by other taxonomically distinct geminiviruses like Tomato yellow leaf curl virus (TYLCV), ACMV and Beet curly top virus (BCTV) [42]. The infectious pseudo-recombination between two distinct bipartite geminiviruses BDMV and ToMoV [20] occurred in spite of divergence at one nucleotide position in the Rep binding motif (iteron) [43]. Pseudo-recombination between Tomato leaf curl New Delhi virus (ToLCNDV) and Tomato leaf curl Gujarat virus (ToLCGV) was reported [44]. This pseudo-recombination resulted in enhanced accumulation of ToLCGV DNA B, despite having a different iteron sequence. Hence, pseudo-recombination can occur between two geminivirus species even with non-identical iterons. Our results show that SLCMV DNA A trans-replicates ICMV DNA B at low levels, in spite of differences in the iteron sequences.

In the same Sengutchi cultivar of cassava, the isolate SLCMV-[SeT1] was non-persistent, whereas the isolate SLCMV-[SeM] was persistent. In cassava plants with mixed infections, recovery from SLCMV facilitated the re-emergence of latent ICMV. This shows that SLCMV suppresses ICMV, which results in the prevalence of SLCMV over ICMV in the cassava fields of the Indian sub-continent. The finding of trans-replication of ICMV DNA B by SLCMV DNA A shows that the dynamics of mixed infections in CMD is very complex.

Elucidation of the basis of recovery of cassava plants from the infection caused by the non-persistent SLCMV-[SeT1] isolate can help in designing a strategy to develop CMD resistance in cassava. The alterations of relative titers of SLCMV and ICMV in cassava under different growth conditions and trans-replication of ICMV DNA B by SLCMV DNA A suggest that both ICMV and SLCMV should be simultaneously targeted in any effort to develop CMD resistance in the Indian subcontinent.

## Figures and Tables

**Table 1:** Sri Lankan cassava mosaic virus (SLCMV) and Indian cassava mosaic virus (ICMV) isolates cloned from the Sengutchi variety of cassava plants collected from the fields of Malappuram (SeM) and Thiruvananthapuram (SeT1).

Geographical Region	Cassava Cultivar	Virus	Isolate	NCBI Accession No.	Length(nt)	Identity
Malappuram	Sengutchi	SLCMV	SeM-DNA-A	KR611577	2756	98% to Kerala 17
						96% to SeT1
		SLCMV	SeM-DNA-B	KR611578	2737	97% to Kerala 4
						98% to SeT1
Thiruvananthapuram	Sengutchi	SLCMV	SeT1-DNA-A	KR611579	2726	99% to Kerala 17
						96% to SeM
		SLCMV	SeT1-DNA-B	KR611580	2739	97% to Kerala 4
						98% to SeM
Re-emergent in Basel	Sengutchi	ICMV	SeT4-DNA-A	KU308385	2735	94% to Kerala 2
		ICMV	SeT4-DNA-B	KU308386	2716	98% to Kerala 6

**Table 2:** Infectivity analysis by agroinoculation of SLCMV-[SeM], SLCMV-[SeT1] and ICMV-[SeT4] partial dimers in *Nicotiana benthamiana* plants.

Component A	Component B	Symptoms (at 14 days post-inoculation (dpi))	Levels of virus DNA
SLCMV-[SeM]		Upward leaf rolling	Low DNA A
	SLCMV-[SeM]	No symptoms	None
SLCMV-[SeM]	SLCMV-[SeM]	Stunting, chlorosis, downward leaf curling	High DNA A and DNA B
SLCMV-[SeT1]		Upward leaf rolling	Low DNA A
	SLCMV-[SeT1]	No symptoms	None
SLCMV-[SeT1]	SLCMV-[SeT1]	Stunting, chlorosis, downward leaf curling	High DNA A and DNA B
ICMV-[SeT4]		Very mild leaf blade curling	High DNA A
	ICMV-[SeT4]	No symptoms	None
ICMV-[SeT4]	ICMV-[SeT4]	Stunting and mild to moderate leaf blade curling	High DNA A and DNA B
SLCMV-[SeM]	ICMV-[SeT4]	Mild leaf blade curling	High DNA A and low DNA B

## Supplementary Materials

The following are available online at www.mdpi.com/1999-4915/8/10/264/s1, **Figure S1**: Infectivity analysis of Sri Lankan cassava mosaic virus (SLCMV)-[SeT1] partial dimers in *Nicotiana benthamiana* plants.

## References

[1] Fauquet C.M., Briddon R.W., Brown J.K., Moriones E., Stanley J., Zerbini M., Zhou X. (2008). Geminivirus strain demarcation and nomenclature. Arch. Virol..

[2] Hanley-Bowdoin L., Bejarano E.R., Robertson D., Mansoor S. (2013). Geminiviruses: Masters at redirecting and reprogramming plant processes. Nat. Rev.Microbiol..

[3] Varsani A., Navas-Castillo J., Moriones E., Hernández-Zepeda C., Idris A., Brown J.K., Zerbini F.M., Martin D.P. (2014). Establishment of three new genera in the family *Geminiviridae*: *Becurtovirus*, *Eragrovirus* and *Turncurtovirus*. Arch. Virol..

[4] Idris A.M., Brown J.K. (2002). Molecular analysis of Cotton leaf curl virus-Sudan reveals an evolutionary history of recombination. Virus Genes.

[5] Legg J.P., Fauquet C.M. (2004). Cassava mosaic geminiviruses in Africa. Plant Mol. Biol..

[6] Legg J.P., Owor B., Sseruwagi P., Ndunguru J. (2006). Cassava mosaic virus disease in East and Central Africa: Epidemiology and management of a regional pandemic. Adv. Virus Res..

[7] Abraham A. (1956). Tapioca cultivation in India. Farm Bulletin No. 17.

[8] Alagianagalingam M.N., Ramakrishnan K. (1966). Cassava mosaic in India. South Indian Hortic..

[9] Saunders K., Nazeera S., Mali V.R., Malathi V.G., Briddon R.W., Markham P.G., Stanley J. (2002). Characterisation of *Sri Lankan cassava mosaic virus* and *Indian cassava mosaic virus*: Evidence for acquisition of a DNA B component by a monopartite begomovirus. Virology.

[10] Hong Y.G., Robinson D.J., Harrison B.D. (1993). Nucleotide sequence evidence for the occurrence of three distinct whitefly transmitted geminiviruses in cassava. J. Gen. Virol..

[11] Patil B.L., Rajasubramaniam S., Bagchi C., Dasgupta I. (2005). Both Indian cassava mosaic virus and Sri Lankan cassava mosaic virusare found in India and exhibit high variability as assessed by PCR-RFLP. Arch. Virol..

[12] Mittal D., Borah B.K., Dasgupta I. (2008). Agroinfection of cloned Sri Lankan cassava mosaic virus DNA to *Arabidopsis thaliana*, *Nicotianatabacum* and cassava. Arch. Virol..

[13] Chellappan P., Vanitharani R., Fauquet C.M. (2004). Short interfering RNA accumulation correlates with host recovery in DNA virus infected hosts and gene silencing targets specific viral sequences. J. Virol..

[14] Vanitharani R., Chellappan P., Fauquet C.M. (2005). Geminiviruses and RNA silencing. Trends Plant Sci..

[15] Rodríguez-Negrete E.A., Carrillo-Tripp J., Rivera-Bustamante R.F. (2009). RNA silencing against geminivirus: Complementary action of posttranscriptional gene silencing and transcriptional gene silencing in host recovery. J. Virol..

[16] Góngora-Castillo E., Ibarra-Laclette E., Trejo-Saavedra1 D.L., Rivera-Bustamante R.F. (2012). Transcriptome analysis of symptomatic and recovered leaves of geminivirus-infected pepper (*Capsicum annuum*). Virol. J..

[17] Hagen C., Rojas M.R., Kon T., Gilbertson R.L. (2008). Recovery from *Cucurbit leaf crumple virus* (Family *Geminiviridae*, Genus *Begomovirus*) infection is an adaptive antiviral response associated with changes in viral small RNAs. Virology.

[18] Chellappan P., Vanitharani R., Ogbe F., Fauquet C.M. (2005). Effect of temperature on geminivirus-induced RNA silencing in plants. Plant Physiol..

[19] Patil B.L., Fauquet C.M. (2015). Studies on differential behavior of cassava mosaic geminivirus DNA components, symptom recovery patterns, and their siRNA profiles. Virus Genes.

[20] Gilbertson R.L., Hidayat S.H., Paplomatas E.J., Rojas M.R., Hou Y., Maxwell D.P. (1993). Pseudo-recombination between infectious cloned DNA components of tomato mottle and bean dwarf mosaic geminiviruses. J. Gen. Virol..

[21] Unseld S., Ringel M., Höfer P., Höhnle M., Jeske H., Bedford I.D., Markham P.G., Frischmuth T. (2000). Host range and symptom variation of pseudorecombinant virus produced by two distinct bipartite geminiviruses. Arch. Virol..

[22] Unseld S., Ringel M., Konrad A., Lauster S., Frischmuth T. (2000). Virus-specific adaptations for the production of a pseudorecombinant virus formed by two distinct bipartite geminiviruses from Central America. Virology.

[23] Fondong V.N. (2013). Geminivirus protein structure and function. Mol. Plant Pathol..

[24] Argüello-Astorga G.R., Guevara-González R.G., Herrera-Estrella L.R., Rivera-Bustamante R.F. (1994). Geminivirus replication origins have a group-specific organization of iterative elements: A model for replication. Virology.

[25] Fujii R., Kitaoka M., Hayashi K. (2006). Error-prone rolling circle amplification: The simplest random mutagenesis protocol. Nat. Protoc..

[26] Hajdukiewicz P., Svab Z., Maliga P. (1994). The small, versatile pPZP family of *Agrobacterium* binary vectors for plant transformation. Plant Mol. Biol..

[27] Ditta G., Stanfield S., Corbin D., Helinski D.R. (1980). Broad host-range DNA cloning system for Gram-negative bacteria: Construction of a gene bank of *Rhizobium meliloti*. Proc. Natl. Acad. Sci. USA.

[28] Jacob S.S., Vanitharani R., Karthikeyan A.S., Chinchore Y., Thillaichidambaram P., Veluthambi K. (2003). *Mungbean yellow mosaic virus*-Vi agroinfection by codelivery of DNA A and DNA B from one *Agrobacterium* strain. Plant Dis..

[29] Chilton M., Currier T.C., Farrand S.K., Bendich A.J., Gordon M.P., Nester E.W. (1974). *Agrobacterium tumefaciens* DNA and PS8 bacteriophage DNA not detected in crown gall tumours. Proc. Natl. Acad. Sci. USA.

[30] Grimsley N., Hohn B., Hohn T., Walden R. (1986). “Agroinfection,” an alternative route for viral infection of plants by using the Ti plasmid. Proc. Natl. Acad. Sci. USA.

[31] Mahajan N., Parameswari C., Veluthambi K. (2011). Severe stunting in blackgram caused by the *Mungbean yellow mosaic virus* (MYMV) KA27 DNA B component is ameliorated by co-infection or post-infection with the KA22 DNA B: MYMV nuclear shuttle protein is the symptom determinant. Virus Res..

[32] Rogers S.O., Bendich A.J., Gelvin S.B., Schilperoort R.A. (1994). Extraction of total cellular DNA from plants, algae and fungi. Plant Molecular Biology Manual.

[33] Hong Y., Stanley J. (1996). Virus resistance in *Nicotiana benthamiana* conferred by African cassava mosaic virus replication associated protein (AC1) transgene. Mol. Plant-Microbe Interact..

[34] Southern E.M. (1975). Detection of specific sequences among DNA fragments separated by gel electrophoresis. J. Mol. Biol..

[35] Balaji V., Vanitharani R., Karthikeyan A.S., Anbalagan S., Veluthambi K. (2004). Infectivity analysis of two variable DNA B components of *Mungbean yellow mosaic virus-Vigna* in *Vigna mungo* and *Vignaradiata*. J. Biosci..

[36] Sunitha S., Shanmugapriya G., Balamani V., Veluthambi K. (2013). *Mungbean yellow mosaic virus* (MYMV) *AC4* suppresses post-transcriptional gene silencing and an *AC4* hairpin RNA gene reduces MYMV DNA accumulation in transgenic tobacco. Virus Genes.

[37] Fasteris—DNA Sequencing Service. https://www.fasteris.com/dna/.

[38] Phytozome v11.0 *Manihot esculenta* v6.1 (Cassava). https://phytozome.jgi.doe.gov/pz/portal.html#!info?alias=Org_Mesculenta.

[39] Li H., Durbin R. (2010). Fast and accurate long-read alignment with Burrows–Wheeler Transform. Bioinformatics.

[40] Rothenstein D., Haible D., Dasgupta I., Dutt N., Patil B.L., Jeske H. (2006). Biodiversity and recombination of cassava-infecting begomoviruses from southern India. Arch. Virol..

[41] Von Arnim A., Stanley J. (1992). Determinants of *Tomato golden mosaic virus* symptom development located on DNA B. Virology.

[42] Dry I.B., Krake L.R., Rigden J.E., Rezaian M.A. (1997). A novel subviral agent associated with a geminivirus: The first report of a DNA satellite. Proc. Natl. Acad. Sci. USA.

[43] Fontes E.P., Gladfelter H.J., Schaffer R.L., Petty I.T., Hanley-Bowdoin L. (1994). Geminivirus replication origins have a modular organization. Plant Cell.

[44] Chakraborty S., Vanitharani R., Chattopadhyay B., Fauquet C.M. (2008). Supervirulent pseudo-recombination and asymmetric synergism between genomic components of two distinct species of begomovirus associated with severe tomato leaf curl disease in India. J. Gen. Virol..

