# Supplementary material for: Emergence of a Latent Indian Cassava Mosaic Virus from Cassava Which Recovered from Infection by a Non-Persistent Sri Lankan Cassava Mosaic Virus
Source: Viruses. 2016 Sep 28;8(10):264. doi: 10.3390/v8100264 (PMC5086600; doi:10.3390/v8100264)
Supplement: Supplementary file 1 [file viruses-08-00264-s001.pdf]

Supplementary Materials: Emergence of a Latent Indian Cassava Mosaic Virus from Cassava Which Recovered from Infection by a Non-Persistent Sri Lankan Cassava Mosaic Virus

Chockalingam Karthikeyan, Basavaprabhu L. Patil, Basanta K. Borah, Thulasi R. Resmi, Silvia Turco, Mikhail M. Pooggin, Thomas Hohn and Karuppannan Veluthambi

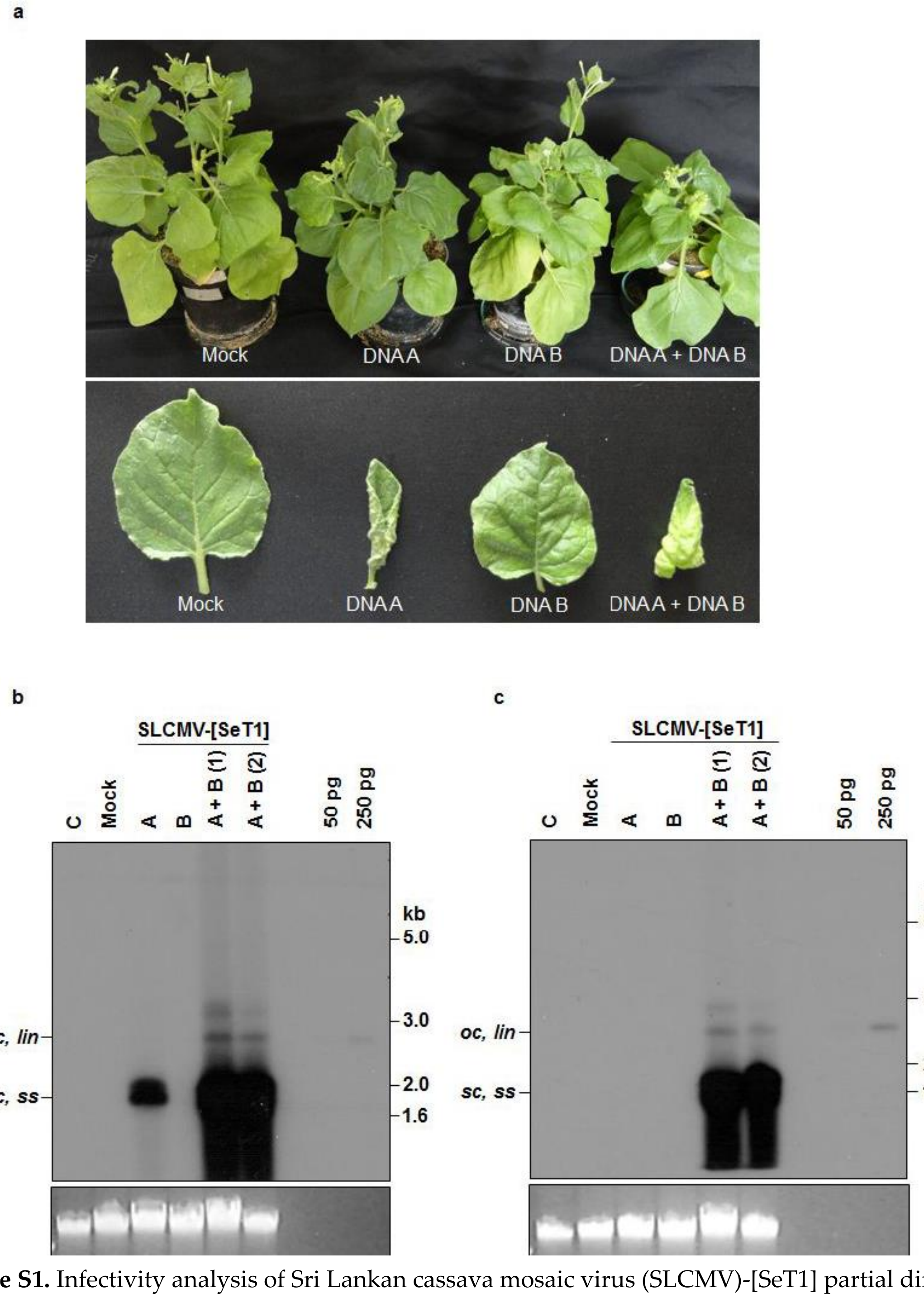

Figure S1. Infectivity analysis of Sri Lankan cassava mosaic virus (SLCMV)-[SeT1] partial dimers in *Nicotiana benthamiana* plants. (**a**) Symptoms displayed by *N. benthamiana* plants agroinoculated with the partial dimers of SLCMV-[SeT1]. Bottom half shows individual leaves of the tested plants. (**b**) Southern blot analysis using SLCMV-[SeT1] DNA-A (without common region [ΔCR]) labelled with

[α-32P]dCTP as the probe. The plasmid pBS-SLCMV-Tv-A digested with PstI (50 pg and 250 pg) was used as the positive control. (**c**) Southern blot analysis using [α-32P]dCTP-labelled SLCMV-[SeT1] DNA-B (ΔCR) as the probe. The plasmid pBS-SLCMV-Ma-B digested with BamHI (50 pg and 250 pg) was used as the positive control. In (**b**) and (**c**), DNA (1 μg) from uninfected plant (C), plant mock infected with the *Agrobacterium tumefaciens* strain Ach5 (Ach5), plant agroinoculated with the partial dimers of DNA-A alone (A), DNA-B alone (B) and plants co-agroinoculated with the partial dimers of DNA-A + DNA-B (A + B) (in duplicates) were loaded in the respective lanes. Positions of different forms of viral DNA-single stranded (*ss*), super-coiled (*sc*), open circular (*oc*) and linear (*lin*) are marked. Ethidium bromide stained high molecular weight plant DNA is shown for loading control at the bottom.
